# Carnitine deficiency presenting with encephalopathy and hyperammonemia in a patient receiving chronic enteral tube feeding: a case report

**DOI:** 10.1186/1752-1947-6-227

**Published:** 2012-07-30

**Authors:** Peter Ling, Douglas J Lee, Eric M Yoshida, Sandra Sirrs

**Affiliations:** 1 Department of Medicine, University of British Columbia, 10th Floor, 2775 Laurel Street, Vancouver, BC V5Z 1M9, Canada; 2 700 W. 57th Avenue, George Pearson Centre, Vancouver Coastal Health, Vancouver, BC V6P 1S1, Canada; 3 Division of Gastroenterology, Diamond Health Care Centre, Vancouver General Hospital, 5153-2775 Laurel Street, Vancouver, BC, V5Z 1M9, Canada; 4 Division of Endocrinology, Diamond Health Care Centre, Vancouver General Hospital, 4th Floor, 2775 Laurel Street, Vancouver, BC V5Z 1M9, Canada

## Abstract

**Introduction:**

Carnitine is an essential cofactor in mitochondrial fatty acid oxidation. Carnitine deficiency results in accumulation of non-oxidized fatty acyl-coenzyme A molecules, and this inhibits intra-mitochondrial degradation of ammonia. Hyperammonemia may lead to encephalopathy. This scenario has been previously reported.

**Case presentation:**

We report the case of a 47-year-old Caucasian man who had sustained a remote motor vehicle accident injury and relied on long-term tube feeding with a commercial product that wascarnitine-free. He was also on phenytoin therapy for control of his chronic seizures. He developed significant acute psychological and behavioral changes superimposed on his chronic neurological impairment. His ammonia level was found to be elevated at 75 to 100μmol/L (normal <35μmol/L). Phenytoin was found to be at a supra-therapeutic level of 143μmol/L (therapeutic range 40–80μmol/L). After adjusting the dose of phenytoin, other pharmacological and hepatic causes of his hyperammonemia and subacute encephalopathy were excluded. His carnitine levels were found to be low. After initiating carnitine supplementation at 500mg twice daily, the patient’s mental status improved, and his ammonia level improved to 53–60μmol/L.

**Conclusion:**

This case illustrates the importance of avoiding carnitine deficiency and anti-convulsant toxicity in tube-fed patients encountered in hospital wards and nursing homes. These patients should have their carnitine levels assessed regularly, and supplementation should be provided as necessary. Manufacturers of enteral feeds and formulas should consider adding carnitine to their product lines.

## Introduction

Carnitine is an essential cofactor synthesized in liver and kidney cells from lysine and methionine. It allows oxidation of long-chain fatty acids [1]. This process is important because unoxidized fatty acid can accumulate and inhibit the urea cycle, resulting in hyperammonemia [1]. Carnitine can also be obtained from dietary sources, with the highest concentrations of carnitine found in red meat and dairy products [2]. Primary carnitine deficiency may result from hereditary defects in the carnitine transport system. Presentations can range from being completely asymptomatic all the way to severe neonatal encephalopathy [3]. In contrast, secondary or acquired carnitine deficiency occurs in adults, especially in those with cirrhotic liver disease and those who receive long-term total parenteral nutrition (TPN) with insufficient carnitine supplementation [3]. Major clinical presentations of carnitine deficiency are hyperammonemic encephalopathy, hypoketotic hypoglycemia, and cardiomyopathy [1]. We present the case of a patient who has been on long-term percutaneous endoscopic gastrostomy (PEG) tube feeding with a carnitine-free feed, manifesting as hyperammonemic encephalopathy. Carnitine deficiency is a rare entity which is frequently not recognized, and this case report demonstrates the importance of awareness of acquired carnitine deficiency in patients receiving TPN.

## Case presentation

Our patient was a 47-year-old Caucasian man with a remote history of head injury and long-term PEG feeding in a nursing home who had developed increasing agitation and confusion. The patient had a closed head injury from a motor vehicle accident sustained 23 years previously. Since then he has had chronic but stable cognitive impairment and resides in an extended care facility. At baseline, he was able to talk and make simple decisions, but not complex ones such as those regarding his care. He is totally unable to perform his activities of daily living (ADLs) and is transferred using a ceiling lift and a two-person assist. He required tracheostomy for breathing and was fed via a PEG tube with Isosource® HN formula (Nestlé Nutrition™, Highland Park, MI, USA) without carnitine (Table 1). He had been stable on tube feeds for many years. His calculated body mass index (BMI) was 24kg/m^2^, and his caloric intake via enteral feeds was 1800kcal/day. His other medical problems included a chronic foot ulcer without active infection and diabetes mellitus. He did not have any history of rhabdomyolysis, cardiac disease, liver disease, or renal insufficiency that would have suggested an underlying genetic defect in fatty acid oxidation. An unexplained acute change in his cognitive and/or psychological function and behavior occurred when he became increasingly confused and drowsy. As the patient had been on chronic phenytoin therapy for years, his phenytoin level was checked and found to be above the therapeutic range at 143μmol/L (therapeutic range 40 to 80μmol/L). His phenytoin dose was adjusted such that, days later, the level was 63μmol/L. Despite the achievement of therapeutic phenytoin concentration, his cognitive changes did not return to baseline. He continued to be delirious with a reversed sleep cycle. The phenytoin was further adjusted such that the level was subtherapeutic at 14μmol/L without improvement in his cognitive status. He did not return to his baseline level of function, being oriented only to person and unable to answer questions.

**Table 1:** Content of Isosource® HN per 100ml (manufactured by Nestlé Nutrition™)

**Measured parameter**	**Value**
Protein	5.3g
Fat	4.2g
Linoleic acid	0.4g
Carbohydrate	15.1g
Energy	210kcal or 500kJ
Vitamin A	330IU
Vitamin D	27IU
Vitamin E	3IU
Thiamine	0.2mg
Riboflavin	0.24mg
Vitamin B_6_	0.27mg
Niacin	2.7g
Vitamin C	20mg
Vitamin B_12_	0.0008mg
Folic acid	0.027mg
Pantothenic acid	1.33mg
Biotin	0.04mg
Vitamin K	0.0062mg
Iron	1.2mg
Calcium	100mg
Phosphorus	100mg
Sodium	110mg
Potassium	180mg
Chloride	110mg
Magnesium	27mg
Iodine	0.016mg
Copper	0.13mg
Manganese	0.2mg
Zinc	1.7mg
Choline	33mg
Selenium	0.007mg
Chromium	0.012mg
Molybdenum	0.008mg

We evaluated him for other causes of confusion, which showed that his hematological profile, electrolytes, and renal function were normal. The glucometer reading was 6.6mmol/L at the time when he was first assessed for his confusion (hemoglobin A1C unknown). His liver biochemistry and International Normalized Ratio remained normal, and the serum total bilirubin was 8μmol/L (normal <18μmol/L ). The serum albumin level was low (29g/L) without any manifestation of hypoalbuminemia. Evidence of myopathy was difficult to assess clinically, given the neurologic compromise due to his remote accident. Creatine kinase and myoglobinuria were not measured at the time. His serum ammonia level, however, was high on multiple measurements taken under ideal conditions, ranging from 75 to 100μmol/L (normal <35μmol/L). The patient was not on any medication associated with hyperammonemia. His enteral feed, Isosource® HN, did not contain supplemental carnitine, and his serum carnitine and plasma amino acid concentrations were measured. His total carnitine was low at 20.5μmol/L (normal 30 to 63μmol/L), and his free carnitine was 17.2μmol/L (normal 22 to 59μmol/L). Acyl carnitine levels in urine were measured before and after carnitine supplementation, and they did not show findings suggestive of an underlying genetic defect of fatty acid oxidation. Plasma concentrations of ornithine (71μM), citrulline (34μM), and arginine (68μM) were normal, so there was no evidence of an undiagnosed urea cycle defect or other aminoacidopathy. Supplementation with carnitine 500mg twice daily via his PEG tube was initiated. His diet was otherwise the same without protein being removed from the diet. The patient’s confusion subsequently improved, and he was restored to his baseline neurological state, free of confusion, agitation, or behavioral disturbances within 1 month of the carnitine supplementation. Repeat serum ammonia concentration after carnitine supplementation decreased promptly to 53 to 60μmol/L.

## Discussion

Conditions associated with hyperammonemic encephalopathy includes organic acidemias, fatty acid oxidation defects, pyruvate metabolism disorders, liver failure, urea cycle defects, and Reye’s syndrome. Iatrogenic causes include adverse drug effects (e.g. valproic acid), TPN with carnitine-free feeds, and transjugular intra-hepatic portosystemic shunts (TIPS) [4]. Rarer causes of hyperammonemia include hyperornithinemia and homocitrullinemia, which are both associated with urea cycle defects [5]. This patient had neither liver disease nor aspirin use associated with Reye’s syndrome. Other than phenytoin, he was not on any medications associated with hyperammonemia. His normal amino acid profile excluded intrinsic urea cycle defect or undiagnosed aminoacidopathy. Given the long term use of carnitine-free feeds, carnitine deficiency is considered in this case. The patient had been clinically stable without hyperammonemia on the same feeding regimen for years. Therefore, the improvement in his encephalopathy was a consequence of carnitine supplementation, instead of secondary to changes in protein content in his diet.

In humans, approximately 75% of body carnitine comes from the diet, such as from meat, fish, and dairy products, and 25% comes from *de novo* biosynthesis from lysine, methionine, and other cofactors [6]. Skeletal and cardiac muscle tissues are the main storage sites for carnitine. Our patient had been on long-term carnitine-free feeds. Repleting all other nutrients without carnitine further raised the tissue requirement for carnitine. In addition, the patient had a low carnitine reserve from his decreased skeletal muscle mass secondary to his physical disability, as evidenced by his creatinine level of 43μmol/L. Endogenous synthesis of carnitine is reduced by undernutrition for iron, protein, niacin, and vitamins C and B_6_, all of which are building blocks for carnitine [2]. Feller *et al*. described 19 nursing home patients who were chronically tube-fed with protein hydrolysate formulae. In half of these individuals, low total and free carnitine levels were detected [2]. These data suggest that elderly patients who are on carnitine-free diets may not have sufficient endogenous synthesis to maintain a normal carnitine level.

One case report [7] described an adult man who, after 1 year of TPN, developed hyperbilirubinemia, hypoglycemia, and generalized muscle weakness. His plasma carnitine was found to be low. Intravenous supplementation with carnitine corrected the plasma carnitine deficiency, hyperbilirubinemia, and hypoglycemia, and it restored the skeletal muscle strength. That patient was also found to have a supratherapeutic level of phenytoin at the time of hyperammonemic encephalopathy [7]. Anti-convulsants such as phenytoin and valproate were reported to lower serum carnitine concentrations [8-10]. Phenytoin, to a lesser extent than valproate, can interfere with tubular reabsorption of carnitine in the kidney [2]. Despite normalization of phenytoin to a therapeutic level, our patient’s cognitive changes did not return to his baseline, and his serum carnitine level was still low. In an otherwise healthy patient, the supratherapeutic range of phenytoin probably does not result in clinical manifestation of carnitine deficiency. In a case such as that of our patient, however, who was already hypocarnitinemic from his feeds, phenytoin toxicity can turn a borderline situation into one that is clinically relevant. His prompt clinical improvement due to carnitine supplementation illustrates his marked carnitine deficiency.

## Conclusions

In healthy adults, carnitine is dispensable because of adequate endogenous synthetic ability. In patients with impaired carnitine synthetic capacity and exposure to low carnitine diets, however, such as the debilitated patients with chronic tube-feeding and chronic use of anti-convulsants, carnitine deficiency is a condition that physicians should keep in mind. In the absence of other causes of hyperammonemia, carnitine deficiency should be considered in the differential diagnosis. These patients should have carnitine levels regularly assessed, and supplementation should be provided as necessary. Manufacturers of enteral feeds and formula should also consider adding carnitine to their products.

## Consent

Written informed consent was obtained from the patient’s next-of-kin for publication of this case report and accompanying images. A copy of the written consent is available for review by the Editor-in-Chief of this journal.

## Competing interests

The authors declare that they have no competing interests.

## Authors’ contributions

SS and EY analyzed and interpreted the patient data regarding encephalopathy secondary to carnitine deficiency. DL was the most responsible physician of the patient described. PL was the major contributor in writing the manuscript. All authors read and approved the final manuscript.
